# Sexual Identity and Birth Cohort Differences in Social Support and Its Link with Well-Being among Sexual Minority Individuals

**DOI:** 10.1007/s10508-022-02366-9

**Published:** 2022-08-18

**Authors:** Chaïm la Roi, David M. Frost, Allen Mallory, Andy Lin, Ilan H. Meyer

**Affiliations:** 1 Swedish Institute for Social Research, University of Stockholm, 106 91 Stockholm, Sweden; 2 Department of Sociology, University of Amsterdam, Amsterdam, The Netherlands; 3 Department of Sociology, University of Groningen, Groningen, The Netherlands; 4 Social Research Institute, University College London, London, UK; 5 Department of Human Sciences, College of Education and Human Ecology, The Ohio State University, Columbus, OH USA; 6 UCLA Office of Advanced Research Computing, University of California in Los Angeles, Los Angeles, CA USA; 7 The Williams Institute, UCLA School of Law, University of California in Los Angeles, Los Angeles, CA USA

**Keywords:** Social support, Well-being, Sexual minorities, Panel data, Sexual orientation

## Abstract

This study examined sexual identity and birth cohort differences in social support and its association with well-being, using a longitudinal national probability sample of 706 cisgender and non-binary sexual minority individuals from the USA. The data allowed for extensive descriptions of perceived social support and support networks across subgroups. Findings demonstrated that sexual identity and birth cohort differences in overall sizes of support networks and levels of perceived social support were small. Furthermore, fixed effects analyses indicated that changes in the size of respondents’ social support networks were not related to well-being, with a one-person change being associated with a .04 SD change in well-being or less, depending on the indicator of well-being being tested. Moreover, changes in perceived social support were only limitedly related to changes in respondents’ well-being, a 1-point change in the scale of perceived social support being associated with a .11 SD change in life-satisfaction. Associations were smaller for overall well-being or psychological distress, the other two indicators of well-being used. Together, these findings could imply that cross-sectional research has overestimated the relevance of social support for the well-being of sexual minority individuals, but also that general social support is insufficiently tailored to the support needs of the sexual minority population.

**Supplementary Information:**

The online version contains supplementary material available at 10.1007/s10508-022-02366-9.

## Introduction

Sexual minority individuals experience lower well-being than their heterosexual counterparts, manifesting in higher levels of suicidality (Peter et al., 2017), depressive symptoms (Marshal et al., 2011; Plöderl & Tremblay, 2015), and other mood and anxiety disorders (Plöderl & Tremblay, 2015; Russell & Fish, 2016). According to the minority stress framework, these health disparities are rooted in the stigmatized social status afforded to sexual minorities in most societies (Meyer, 1995, 2003). More precisely, the framework outlines a number of stigma-related stressors that sexual minority people experience because of prejudice and discrimination, which in turn put them at greater risk for lower well-being than their heterosexual peers.

To obtain a more complete picture of the well-being of sexual minority individuals, researchers ought to also pay attention to factors that make them thrive (Meyer, 2015). Social support may be one such factor. Decades of research have shown social support to be positively associated with well-being (Holt-Lunstad & Uchino, 2015; Kawachi & Berkman, 2001; Umberson & Karas Montez, 2010). Moreover, studies suggest that social support from families, friends, and peers is associated with healthy psychosocial adjustment in sexual minority individuals (e.g., Fingerhut, 2018; Sheets & Mohr, 2009; Watson et al., 2019).

There are many sources of variability among sexual minority populations. Studies comparing bisexual individuals to lesbian women and gay men have shown that bisexual men and women have lower well-being, reflected by increased risks for suicidality (Marshal et al., 2011), depressive symptoms (Ross et al., 2018), and other mood and anxiety disorders (Bostwick et al., 2010). In addition, especially among younger sexual minority individuals, new sexual identity labels proliferate, with more people than in previous generations identifying themselves as pansexual or queer (e.g., Morandini et al., 2017). Furthermore, although sexual identity is a stable, trait-like construct for many people, a non-trivial proportion of the population experiences a change in sexual identity over time because of developmental processes, experiences of sexual fluidity, and dynamics in interpersonal and sociocultural contexts (Campbell et al., 2021; Ott et al., 2011). Whether non-LGB sexual minority individuals and sexual minority individuals that change sexual identity labels over time differ from lesbian, gay and bisexual individuals in access to social support and its importance for well-being, remains scarcely studied, however. Birth cohort may be another important source of variation. Greater societal acceptance and legal changes in laws such as the abolition of sodomy laws and legalization of same-sex marriages has impacted the social environment of sexual minorities (Meyer et al., 2021). These societal changes may have led to better access to social support in younger birth cohorts of sexual minority individuals and changes in the importance of support for well-being.

Therefore, the aim of this paper is to assess the patterns of social support and implications for well-being in diverse sexual minority groups. Using a national probability sample of sexual minority individuals from the USA, we explored whether groups defined by sexual identity and birth cohort differed in access to social support (*levels*), as well as in how social support affected well-being (*function*). Both perceived social support and support networks were used as social support indicators. In doing so, this paper makes several contributions. First, most studies on the effect of social support on well-being among sexual minority individuals relied on community samples, which limits generalizability of findings. The national probability sample employed here alleviates these concerns. Furthermore, longitudinal data allowed us to estimate how within-individual changes in social support over time predicted within-individual changes in well-being, rather than relying on between-individual or cross-sectional analyses. Moreover, this study allows for an extensive exploration of the levels and functions of multiple dimensions of social support, while acknowledging variation within sexual minority groups.

### The Importance of Social Support for Well-Being

The link between social support and well-being has inspired decades of research (Berkman et al., 2000; Cohen & Wills, 1985; House et al., 1988; Kawachi & Berkman, 2001; Uchino et al., 2012). The literature has investigated two potential pathways linking social support to well-being: social support as a buffer of the impact of stress on adverse health outcomes and social support as a direct predictor of well-being (Cohen & Wills, 1985; Kawachi & Berkman, 2001). In this study, we focus on this direct effect of social support on well-being, which has been argued to have been more robustly corroborated than the stress-buffering effect (Lakey & Orehek, 2011).

Several arguments have been offered as to why social support would be conducive to well-being (Thoits, 2011). For instance, the existence of supportive relationships may produce positive psychological states, including a sense of belonging, security, and recognition of self-worth (Baumeister & Leary, 1995; Thoits, 1985). Building on these notions, relational regulation theory (Lakey & Orehek, 2011) claims that social support produces well-being through affective, day-to-day interactions or shared activities with valued others. Additionally, social support by significant others may help people capitalize on opportunities for exploration and learning by receiving help or being supported in efforts and initiatives, with positive effects for well-being (Feeney & Collins, 2015; Mikulincer & Shaver, 2009).

### Support Networks, Perceived Social Support, and Mental Health

Social support may thus be beneficial for well-being for several reasons, but not all aspects of social support may be equally important in producing well-being. Studies have repeatedly found that subjective indicators of social support such as perceived social support (Zimet et al., 1988) are better predictors of well-being than the actual size or structure of social support networks (Chu et al., 2010; Cohen & Wills, 1985), which are most often operationalized as the size of people’s overall social networks. These findings have usually been interpreted as indicating that subjective aspects of social support matter more for well-being than the actual structure of support networks. However, it could also mean that the overall size of people’s social networks is a bad proxy for the number of contacts one can rely on for social support and that for studying their effect on well-being, social support networks should be measured directly. Research doing so is scare, but the few studies available have reported positive effects of the size of individuals’ social support networks on well-being, over and above the effect of subjective aspects of social support (Green et al., 2012; Thomas et al., 2016; Wang, 2014). This suggests that the importance of size and structure of social support networks for well-being may have been underestimated in existing research.

### How Has the Effect of Social Support on Well-Being Been Tested in Sexual Minority Populations?

The effect of social support on the well-being of sexual minority individuals has been examined extensively in recent years. The majority of studies suggest that social support is beneficial for the well-being of sexual minority individuals (e.g., Lampis et al., 2021; Lyons et al., 2017; McConnell et al., 2015). Moreover, research employing general population samples suggest that a comparative lack of social support partly explains sexual orientation mental health disparities (Bränström, 2017; Perales & Campbell, 2020; Perales & Todd, 2018). Although these studies underline the importance of examining whether different aspects of social support affect the well-being of sexual minority individuals, most studies unfortunately employed cross-sectional data, using non-probability samples (Lyons et al., 2015; Sattler et al., 2016; Toplu-Demirtaş et al., 2018). Non-probability samples may return unrepresentative estimates of the importance of social support for the well-being of sexual minority individuals. Cross-sectional research designs could, among other things, suffer from omitted variable bias. This means that unless all relevant confounders are accounted for, the estimated effect of social support on well-being will be biased.

One efficient way to circumvent omitted variable bias is to employ so-called fixed effects models using panel data (Allison, 2009; Vaisey & Miles, 2017). Through within-person centering, this technique isolates the effect of within-person change in social support on within-person change in well-being. Thereby, all possible time-constant confounders of the link between social support and well-being (with a time-constant effect) are automatically accounted for (Allison, 2009).

A small number of studies used representative data to test the effect of social support on well-being in sexual minority individuals, either cross-sectionally (Cain et al., 2017; Krueger & Upchurch, 2020) or over time, yet without employing a panel design (Al-Khouja et al., 2021). These studies report beneficial effects of social support on well-being. Another set of studies instead used non-probability samples but with panel designs and hierarchical linear models for analyses (Birkett et al., 2015; Liu & Mustanski, 2012; McConnell et al., 2016). In such models, effects of time-varying variables represent a mix of within-person and between-person associations, unless these variables are explicitly centered by each individual (Enders & Tofighi, 2007; Snijders & Bosker, 2012; Vaisey & Miles, 2017). Thereby, hierarchical linear models run the risk of biased estimation by omitting confounders at the between-person level.

In sum, there is a need for research using probability samples and panel data designs to produce population-representative estimates of the within-person association between social support and well-being among sexual minority individuals.

### Variations in Social Support within the Sexual Minority Population

Most research on sexual identity contrasts within the sexual minority population has focused on differences between bisexual individuals on the one hand and lesbian women and gay men on the other (Rust, 2002). Bisexual individuals tend to report somewhat lower levels of well-being than lesbian and gay men and women (e.g., Ross et al., 2018). Biphobia (e.g., the prejudice that bisexuality is not a “valid” sexual identity and merely a transitory stage in the development of a lesbian or gay identity), higher levels of identity concealment, and lower levels of LGBT community connectedness have been put forth as explanations of this difference (Sarno & Wright, 2013). These factors may also leave bisexual individuals deprived of resources and opportunities for coping and social support in comparison with gay and lesbian men and women (Kwon, 2013; Meyer, 2015). In line, a recent study using Canadian data found that bisexual individuals had lower levels of social support than lesbian and gay men and women (Stinchcombe et al., 2020). Having said that, other recent studies reported no substantial differences in access to social support between lesbian and gay individuals on the one hand, and bisexual individuals on the other (Cain et al., 2017; Ehlke et al., 2020), or even found that bisexual individuals had slightly better access to social support than their lesbian and gay counterparts (Wang et al., 2021a, 2021b).

Social support differences between people identifying as lesbian or gay and non-LGB sexual minority individuals (e.g., queer, pansexual) remain virtually unstudied. Research on sexual minority individuals not identifying as lesbian, gay, or bisexual is a recent endeavor, focusing mostly on mapping diversity in terms that (sexual minority) people use for describing their sexual identity (e.g., Galupo et al., 2015; White et al., 2018). A study by McConnell et al. (2015) reported similar level of social support for sexual minority individuals identifying as lesbian or gay, bisexual, and “something else” (Table 1). Other research, however, has shown that sexual identity change over time may be a risk factor for decreased well-being, arguably because lacking a stable social (sexual) identity may deprive individuals who change identity labels of access to social support and support networks in sexual minority communities (Everett, 2015).

**Table 1:** Correlations and descriptive statistics

Variables		1.	2.	3.	4.	5.	6.
1	Cantril	.					
2	Life satisfaction	.68	.				
3	K6	− .53	− .56	.			
4	Perceived social support	.32	.43	− .23	.		
5	Everyday social support network	.17	.25	− .16	.31	.	
6	Major social support network	.14	.22	− .16	.26	.47	.

		1.	2.	3.	4.	5.	6.
	*M*	6.35	4.24	8.13	5.26	6.02	3.29
	*SD*	1.72	1.68	5.11	1.16	5.01	2.99
	Range	0–10	1–7	0–24	1–7	0–30	0–17
	Percent missing	7.44	7.72	8.14	9.14	8.29	8.85

Groups (*n* individuals/%)							
	*Sexual identity*						
	Lesbian/Gay	385	54.5%				
	Bisexual	166	23.5%				
	Something else	50	7.1%				
	Changed labels	105	14.9%				
	*Cohort*						
	Younger (1990–1997)	275	39.0%				
	Middle (1974–1981)	157	22.2%				
	Older (1956–1963)	274	38.8%				

Based on unimputed combined wave 2-wave 3 data for 706 respondents in analytical sample

It is difficult to predict if the increasing social and legal acceptance of sexual diversity has led to birth cohort differences in social support. On the one hand, growing up in a society that is more welcoming to sexual minority individuals might make it easier to fulfill social support needs. On the other hand, similar levels of minority stress and psychological distress in older and younger generations of sexual minority individuals suggest that improved social and legal climates may not necessarily translate to improvements in the lived experiences of sexual minority individuals (Liu & Reczek, 2021; Meyer et al., 2021). Moreover, improved social and legal climates regarding sexual diversity might have had period effects on access to social support for all cohorts of sexual minority individuals. Suggestive evidence in line with a period effect is provided by a recent study showing that older US sexual minority individuals have social networks of similar size as heterosexual individuals of the same age (Hsieh & Wong, 2020). To the best of our knowledge, cohort differences in social support within the sexual minority population have not been studied.

### Variation in the Effect of Social Support on Well-Being in the Sexual Minority Population by Sexual Identity and Birth Cohort

Sexual identity groups and birth cohorts may thus have unequal access to social support. In addition, the importance of social support in producing well-being may differ across these groups. As such, subgroup differences in well-being may not only be brought about by differential access to social support, but also by differences in the effect of social support on well-being.

Theory allows for different expectations when studying which groups benefit most from social support. Some argue that the beneficiality of social support is demand-driven, with individuals most in need of social support standing to gain most from it (Melrose et al., 2015). This would imply that the importance of and access to social support are inversely related. Or, in other words, that there is decreasing marginal utility in access to social support. Following this line of argumentation, social support may be most beneficial for non-LG sexual identity groups and older birth cohort, for whom access to social support may be relatively scarce.

Other theoretical viewpoints, such as the resource multiplication hypothesis, would however lead to opposite expectations. This hypothesis states that having access to resources enables people to better reap the benefits that other resources provide (Stienstra et al., 2021). This argument fits well with the assumption that social support is conducive for well-being by helping people capitalize on opportunities (Feeney & Collins, 2015; Mikulincer & Shaver, 2009), as this implies that social support is most beneficial for those to whom opportunities to thrive are more often provided. Together, these arguments imply that social support will be more beneficial for those identity groups and birth cohorts within the sexual minority population that are perceived as relatively less deprived, such as lesbian and gay individuals when talking about subgroups in terms of sexual identity, and younger birth cohorts.

Sexual identity and birth cohort differences in the importance of social support for well-being have been scarcely studied empirically, and the few studies examining this topic do not paint a clear picture. Wattson et al. (2019) report variation in the effects of social support between lesbian/gay and bisexual adolescents, but the direction of differences varied depending on the sources of social support (friends, family, teachers, or classmates) studied. Stinchcombe et al. (2020) did not find the effect of social support on depressive symptoms to differ substantially between lesbian/gay and bisexual respondents. In sum, both theory and empirical results allow for different expectations regarding which sexual minority identity groups and birth cohorts will benefit most from the provision of social support.

### The Present Study

Using longitudinal, nationally representative data from three birth cohorts of sexual minority individuals, this study addressed two aims. Aim 1 was to describe the levels and sources of social support and assess differences and similarities in social support patterns among sexual identity and birth cohort groups. Based on earlier literature describing bisexual individuals as a group facing “double stigma” in that they may face stigma from both the sexual minority community and society at large (Bostwick et al., 2010), we anticipate that bisexual individuals have less access to social support than lesbian women and gay men. We also expect this to be the case for non-LGB individuals and sexual minority individuals reporting change in their sexual identity over time when comparing their access to social support to that of lesbian and gay individuals, although the literature base to derive these latter expectations from is small.

To grasp the implications of these subgroup differences in levels of social support, Aim 2 was to test the association between social support and well-being across sexual identity and birth cohort subgroups. Theory and available empirical evidence allowed for expectations in different directions in terms of sexual identity and birth cohort differences in the link between social support and well-being. Therefore, Aim 2 analyses were more exploratory.

Analyses employed multiple measures of social support, looking at both subjective indicators of social support in the form of perceived social support and social support networks, and multiple indicators of well-being. In addition to effects of overall sizes of support networks and perceived support, we also conducted analyses for social support specifically from friends and families, which have been identified as important sources of support for sexual minority individuals in earlier research (e.g., Watson et al., 2019).

## Method

### Participants

We used data from the Generations Study, a three-year longitudinal study designed to examine health and well-being across three cohorts of sexual minority individuals in the USA (Meyer et al., 2020). These birth cohorts were purposefully selected to study identity, stress, health outcomes, and health care and service utilization among sexual minorities from three generations of adults who came of age during distinctly different historical contexts. The oldest cohort came of age during a period labeled “pride” (born 1956–1963), a time when homosexuality was still considered a mental disorder and sodomy was illegal in many states. But, sexual minority people in this era began early efforts to cultivate pride in their communities. It was an era when sexual minority (a “gay” identity) was born as a modern civil rights movement. The second generation, labeled “visibility” (born 1974–1981), came of age during a period when public discourse was centered around the AIDS epidemic, but also a period characterized by a significant strengthening of LGBT institutions including political activist organizations, community health centers, and other community development. The youngest generation was labeled “equality” (born 1990–1997). During the teenage years of this cohort, public discourse had shifted to a one about the equality of sexual minorities and demands (and some successes) regarding their cultural inclusion.

Participants were recruited by Gallup using a national probability sample of adults in the USA and a 2-step recruitment procedure by phone. First, LGBT individuals were identified with the following question: “Do you, personally, identify as lesbian, gay, bisexual, or transgender?” Then, in the second step, respondents who were identified as LGBT were assessed for eligibility for participation. Respondents were eligible if they identified as sexual minorities (lesbian, gay, bisexual, queer, etc.) but not transgender (transgender individuals were recruited into a sister study TransPop (http://www.transpop.org), not reported here); if they were in the groups targeted for the three cohorts under investigation (years of birth 1956–1963, 1974–1981, or 1990–1997); were Black, Latino, or White (or bi- or multiracial including one of these groups); completed at least 6th grade; and spoke English well enough to conduct the phone recruitment interview. Eligible respondents who agreed to participate were sent a survey questionnaire to complete by self-administration via a web link or printed questionnaire, respectively. In total, 366,644 participants were screened for inclusion between March 28, 2016 and March 30, 2017. Of them, 3.5% were identified as LGBT and 27.5% of them were eligible for participation. Of those eligible, 81% agreed to participate in the survey and of those, 48% completed the survey. The final baseline sample size was 1,331. Following this baseline interview, respondents were scheduled to complete two follow up surveys, one year apart, at wave 2 and wave 3. More information about the study methods is available at http://www.generations-study.com/methods.

This study focused on social support. As social support networks were only measured at waves 2 and 3, all analyses were conducted on combined wave 2-wave 3 data, which included entries from respondents participating in both waves 2 and 3 (*n* = 616) and respondents participating in wave 3 only (*n* = 91). In this group, one respondent identified as straight/heterosexual and was therefore excluded from analyses. The final analytical sample thus consisted of 706 respondents.

### Measures

#### Well-Being

*The Cantril scale* is a single-item measure of overall well-being: “Please imagine a ladder with steps numbered from zero at the bottom to ten at the top. The top of the ladder represents the best possible life for you, and the bottom of the ladder represents the worst possible life for you. On which step of the ladder would you say you personally feel you stand at this time?” The Cantril scale has shown to be a valid measure of well-being (Levin & Currie, 2014).

*Satisfaction with Life* is a 5-item scale for measuring global life satisfaction (Diener et al., 1985). Respondents rated the following items on a scale from 1 (*strongly disagree)* to 7 *(strongly agree)*: *“*In most ways, my life is close to my ideal”; “The conditions of my life are excellent”; “I am satisfied with life”; “So far I have gotten the important things I want in life.”; “If I could live my life over, I would change almost nothing.” The final scale comprised the mean score on all five items. Internal consistency was high (Wave 2 *α* (W2 *α*) = 0.92; Wave 3 *α* (W3 *α*) = 0.92).

*K6* is a six-item self-report questionnaire measuring psychological distress (Kessler et al., 2003). Respondents were asked “During the past 30 days, about how often did you feel…”, “Nervous/Hopeless/Restless or fidgety/So depressed that nothing could cheer you up/That everything was an effort/Worthless.” The mean score on all six items comprised the final scale. The response scale ranged from *All of the time* (0) to *None of the time* (4) (W2 *α* = 0.89; W3 *α* = 0.89).

#### Social Support

*Perceived social support* is a 12-item self-report measure of subjectively assessed social support (Zimet et al., 1988). Respondents could indicate that they *very strongly disagree* (1) to *very strongly agree* (7) on items such as “There is a special person who is around when I am in need”; “My friends really try to help me”; “My family is willing to help me make decisions.” The mean response to all items was used as an indicator of overall perceived social support. Two subscales account for perceived social support from family and friends (W2 *α* = 0.91; W3 *α* = 0.92).

*Size everyday social support network* (adapted from Frost et al., 2016)*.* Respondents were asked: “For these next questions, first please write down for yourself a list of the initials or first names of as many people you could count on for everyday support over the past year. By everyday support, we mean things like when you need to discuss worries, share happiness, help with household chores, or someone to confide in or to share social activities with. How many people did you list?” This was followed by: “Thinking only about the people you listed in the prior question, how many of them are…” (a) Your family (other than your spouse), (b) Your spouse, (c) Your close friends, (d) Your friends/acquaintances, (e) Volunteer/paid worker, (f) Other. The total number of persons listed and numbers of persons listed for family and close friends were used as measures of the size of respondents’ everyday social support network and the size of their everyday social support network consisting of family and friends. As the raw measures contained extreme outliers to the right, entries in the highest percentile were top coded to reduce the impact of outliers in analyses.

*Size major social support network* was operationalized in the same way as everyday social support, except that respondents this time were primed to think of people they could rely on for major social support, which was defined as follows: “By major support, we mean things like when you need to borrow a large sum of money (e.g., several hundred dollars) for an emergency such as rent or a medical emergency; when you need help making important decisions about your life such as decisions about your family, money or health; and when you need someone to take care of you or help you out when you’re sick.”

*Sexual identity*. This was measured using the following question: “Which of the following best describes your current sexual orientation?”, with response options being Straight/heterosexual, Lesbian, Gay, Bisexual, Queer, Same gender-loving, and Other, followed by a fill-in response option that was later coded as “pansexual, asexual and other.” Sexual identity was coded as Lesbian/Gay, Bisexual, and grouping additional groups under the label Something else due to low *n*. We categorized respondents that gave varying responses to the sexual identity question between waves as Changed labels. The *Lesbian/Gay* sexual identity group was used as the reference group in regression analyses.

*Birth cohort*. The study collected data on three theoretically targeted birth cohorts (Krueger et al., 2022), here labeled Younger (born 1990–1997), Middle (born 1974–1981), and Older (born 1956–1963).

### Data

#### Attrition

Of the 1331 respondents included in the wave 1 sample, 894 (67%) participated again at wave 2, and 707 (53%) at wave 3. Therefore, we tested whether attrition was related to our study variables. Attrition between wave 1 and wave 3 was not related to the size of respondents’ wave 2 social support network or levels of perceived social support. There was an association with wave one well-being, however. Compared to participants at follow-up, dropouts reported somewhat lower Cantril scale levels (Odds-ratio (*OR*) = 0.92, *95% CI* [0.86, 1.00], *p* = .044), life satisfaction (*OR* = 0.89, *95% CI* [0.82, 0.96], *p* = .005), and higher levels of depressive symptoms (*OR* = 1.06, *95% CI* [1.03, 1.08], *p* < .001) at wave 1. Furthermore, respondents identifying as bisexual (*OR* = 1.37, *95% CI* [1.00, 1.86], *p* = .047) were somewhat more likely to drop out than respondents identifying as lesbian or gay, and respondents from the youngest (*OR* = 2.00, *95% CI* [1.50, 2.68], *p* < .001) and middle birth cohort (*OR* = 1.75, *95% CI* [1.24, 2.47], *p* = .001) were more likely to drop out than respondents from the older birth cohort. To prevent biased estimates due to dropout, presented analyses employed longitudinal sampling weights that accounted for attrition when reweighting back to the population (Krueger et al., 2022).

#### Partial Non-Response

In addition to attrition, we report percentages of partial non-response for observations included in the analytical sample. These percentages are displayed in Table 1, ranging between 7.72% (life satisfaction) and 9.14% (perceived social support). In order to minimize potential bias due to missingness in multivariate models, we multiply imputed missing data using chained equations (Van Buuren & Groothuis-Oudshoorn, 2011; White et al., 2011). Twenty imputed datasets were created.

### Analysis Plan

After describing support networks and levels of perceived social support using histograms, we estimated how social support varied by sexual identity group and birth cohort using negative binomial (for support networks) and linear (for perceived social support) regressions. Coefficients for negative binomial regressions were presented as incidence rate ratio’s (IRRs), and those for linear regressions were y-standardized such that group differences can be interpreted as Cohen’s *d*’s (Cohen, 1992).

To assess whether social support had a positive impact on well-being and whether this impact varied by sexual identity group, we conducted fixed-effects regression analyses (Allison, 2009) (identical to so-called change score models for two waves of data Allison, 2009), regressing within-person changes in well-being on changes in social support. The main strength of our fixed effects analysis is its ability to cancel out potential confounding by omitted between-person variables by focusing solely on within-person variance (Allison, 2009; Gardiner et al., 2009; Vaisey & Miles, 2017). This strength may be of particular importance when analyzing the link between social support and well-being, as differences *between persons* on both these concepts may be influenced by a myriad of third factors, some of which may be hard to measure (comprehensively), such as personality traits (House et al., 1988).

To explore whether estimates of the link between social support and well-being may be biased by omitted confounders at the between-person level, we compared estimates from fixed effects regressions with those from random effects regressions (equivalent to “random intercept models” within the multilevel modeling literature), which provide more efficient estimates than fixed effects through employing both within- and between-person variation, yet result in biased estimates when the assumption of no omitted confounders at the between-person level is violated (e.g., Vaisey & Miles, 2017).

As a prerequisite for conducting fixed effects analyses, we tested for evidence of substantial within-person variance across survey waves in both predictors and outcomes. Estimates of intraclass correlations suggested that there was substantial within-person variance in both social support and well-being, with intraclass correlations ranging between .61 (size major social support network) and .36 (depressive symptoms). About 53 and 38 percent of respondents reported a change of at least two persons in the size of their everyday and major social support networks, respectively, and 25 percent of respondents reported a change of 1 scale point or more in perceived social support. As 60 and 85% of respondents had everyday and major social support networks consisting of 5 or fewer people and as the SD of the perceived social support scale was about one scale point, between-wave changes in social support thus were substantial. All in all, the data thus allowed for fixed effects analyses. In addition to estimating main effects of social support on well-being, we tested whether effects of social support on well-being varied by sexual identity by interacting effects of social support with sexual identity dummies and evaluating contrasts between conditional effects of social support by sexual identity group. To optimize interpretability of findings, results were y-standardized.

Sampling weights were employed across all presented analyses. All analyses were conducted in Stata 16. Figures were prepared in R.

## Results

### Descriptive Statistics

Descriptive statistics of and point estimates for pairwise correlations between study variables are reported in Table 1. Correlations between well-being outcomes were moderately high (between .53 and .68 in absolute size). Correlations between perceived social support and the size of respondents’ social support networks were around .2–.3, highlighting that the amount of support respondents perceived correlated only moderately with the size of their support networks. Correlations between well-being and social support indicators were small to moderate, ranging between .14 and .43 in absolute size. The group of respondents identifying as lesbian or gay (*n* = 385) was larger than those identifying as bisexual (*n* = 166), “something else” (*n* = 50), or people reporting different sexual identities across survey waves (*n* = 105). There were somewhat fewer respondents from the middle (*n* = 157), than from the younger (*n* = 276) and older (*n* = 274) birth cohorts.

### Aim 1: Patterns of Social Support

**Fig. 1:**
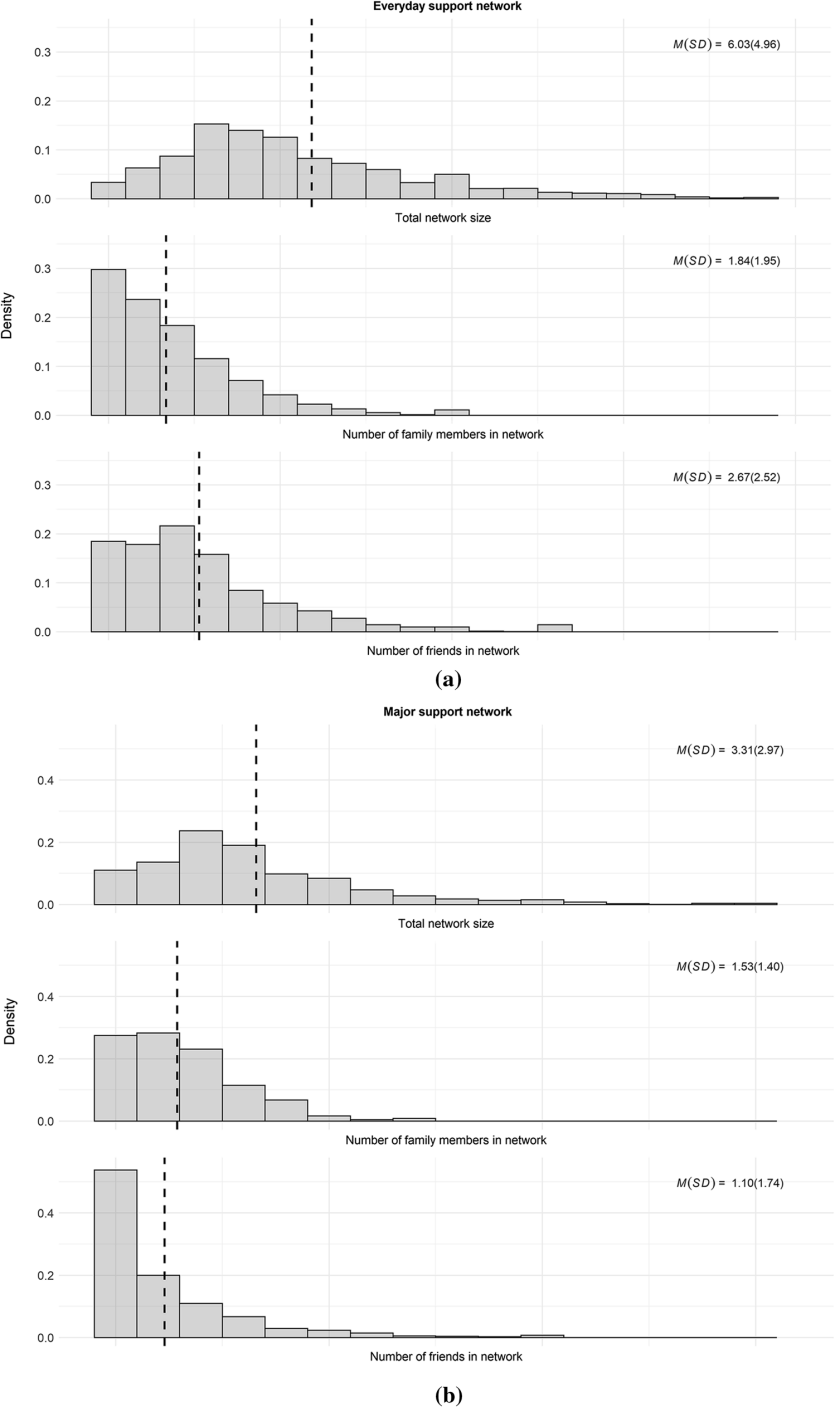

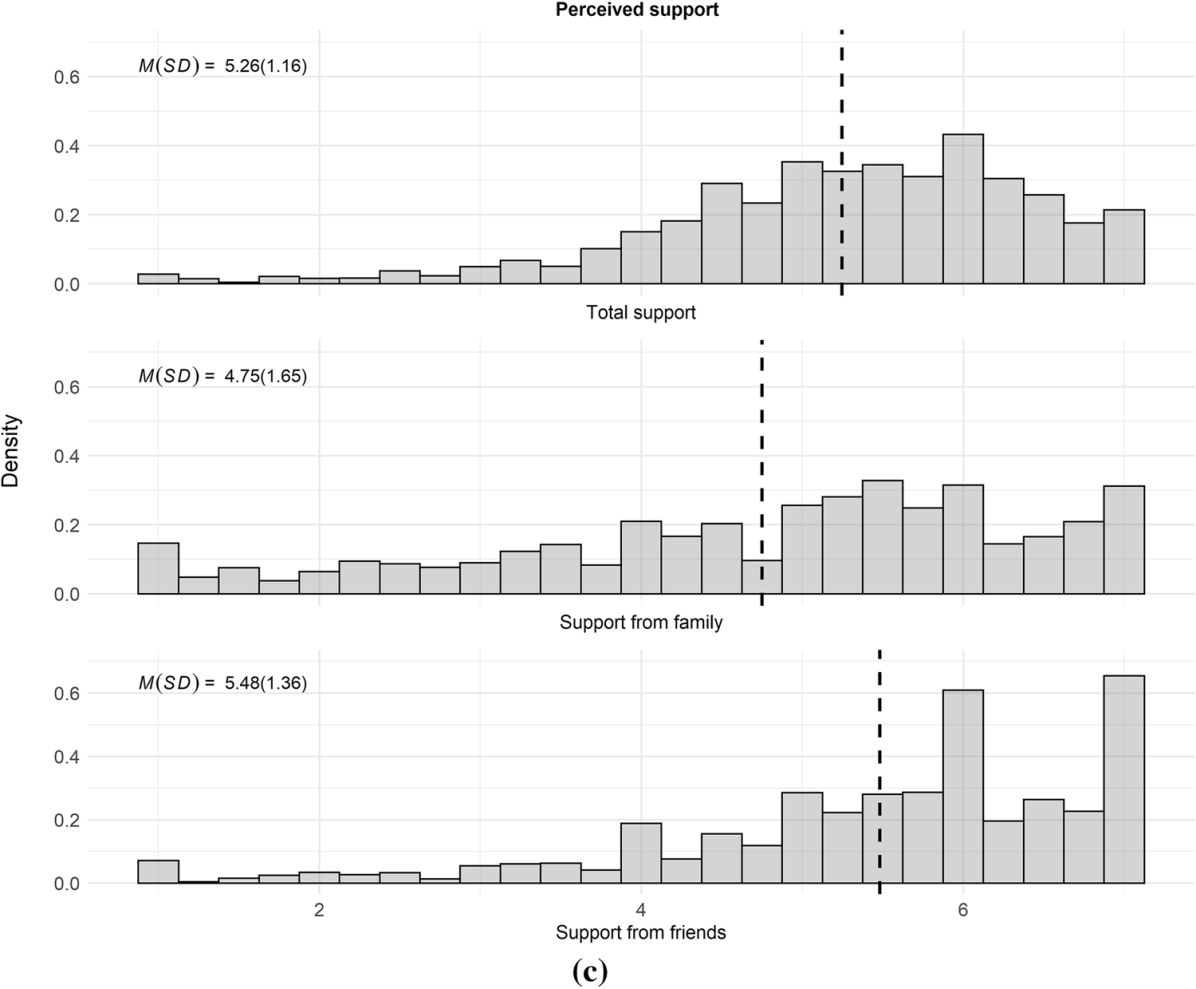
**a** Histograms of everyday social support networks. **b** Histograms of major social support networks. *Notes* Dashed vertical lines display means. Small number of extreme outliers on right-hand side outside graph area. MI data, weighted. *N*_*obs*_ = 1412, *N*_*persons*_ = 706. **c** Histograms of perceived social support. *Note* Dashed vertical lines display means. MI data, weighted. *N*_*obs*_ = 1412, *N*_*persons*_ = 706

Figure 1a–c provides a description of social support networks and levels of perceived social support for the sample. The distribution of support networks was skewed, indicating that most respondents relied on a small number of people for social support, whereas a small minority of respondents had access to many others for this. For everyday social support, counting on 3, 4, or 5 others was most common. For major support, relying on 2 people was most common.

We also assessed the relative importance of family versus friends for social support (Fig. 1a, b). Everyday social support networks consisted of fewer family members (*M/(SD)* = 1.84(1.95)), than friends (*M/(SD)* = 2.67(2.52), *95% CI difference* [− 1.07, − 0.59]). The reverse was true of major social support networks, which consisted of slightly more family members (*M/(SD)* = 1.53(1.40)) than friends (*M/(SD)* = 1.10(1.74), *95% CI difference* [0.24, 0.60]).

#### Group Differences in Social Support

Several sexual identity group differences stood out in the composition of social support networks in terms of friendship and family ties (full results available in Tables A1 an A2 of the Online Supplementary). Compared to lesbian and gay respondents, bisexuals reported almost a quarter fewer friends to rely on for both everyday (*IRR* = 0.78, *95% CI* [0.64, 0.95]) and major social support (*IRR* = 0.76, *95% CI* [0.54, 1.07]). The composition of everyday social support networks of individuals categorized as “something else” and those who changed labels was similar to that of lesbian and gay individuals, but the composition of their major support networks differed somewhat: Respondents identifying as “something else” had somewhat fewer friends to rely on for major support than lesbian and gay individuals, (*IRR* = 0.76, *95% CI* [0.45, 1.27]), whereas the opposite held for individuals changing labels (*IRR* = 1.20, *95% CI* [0.86, 1.66]).

Birth cohort differences were discovered in the composition of support networks, too. Compared to respondents of the youngest birth cohorts, respondents from both the middle and older cohorts relied somewhat less on family for both everyday (*IRR middle-young* = 0.75, *95% CI* [0.59, 0.95]; *IRR older-young* = 0.78, *95% CI* [0.59, 1.04]) and major social support (*IRR middle-young* = 0.76, *95% CI* [0.62, 0.92]; *IRR older-young* = 0.68*, 95% CI* [0.56, 0.84]).

**Fig. 2:**
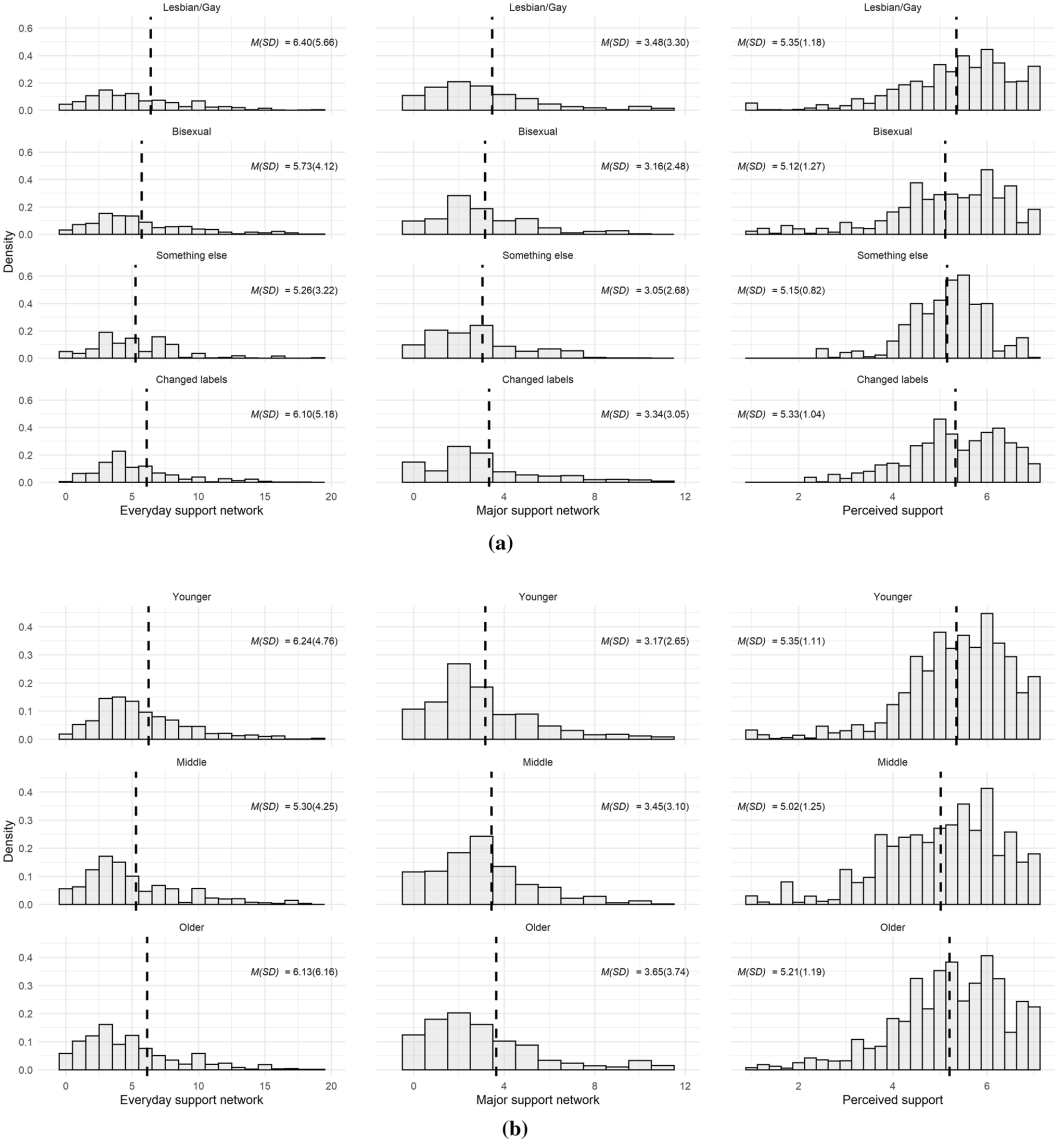
**a** Histograms of social support networks and perceived social support by sexual identity group. **b** Histograms of social support networks and perceived social support by birth cohort. *Note* Vertical dashed lines display group means. MI data, weighted. *N*_*obs*_ = 1412, *N*_*persons*_ = 706

However, these compositional differences did not translate into substantial differences in the overall size of social support networks and levels of perceived social support across sexual identity and birth cohort groups were modest. As summarized in Table 2 and Fig. 2a, b, overall sizes of support networks were very similar across sexual identities and birth cohorts. Regarding sexual identity, group differences in everyday social support networks were largest between lesbian/gay individuals [*M(SD)* = 6.40(5.66)] and those identifying as “something else” [*M(SD)* = 5.26(3.22)]. Differences in average size of major social support networks were less than 0.5 persons across sexual identity groups. Regarding birth cohort, group differences in everyday social support networks were largest between the younger [*M(SD)* = 6.24(4.76)] and middle cohort [*M(SD)* = 5.30(4.25)]. As with sexual identity groups, birth cohort differences in the average size of major social support networks were less than 0.5 persons. Group differences in perceived social support were modest too, although individuals categorized as bisexual [*M(SD)* = 5.12(1.27)] and “something else” [*M(SD)* = 5.15(0.82)] displayed somewhat lower levels of perceived social support than lesbian and gay individuals [*M(SD)* = 5.35(1.18)] (see also Table 2). Regarding bisexual individuals, these differences were mostly brought about by lower levels of perceived support from friends (*b* = − 0.30, 95% CI [− 0.55, − 0.06]), whereas individuals categorized as “something else” perceived less support from family than gay and lesbian men and women (*b* =  − 0.34, 95% CI [− 0.68, − 0.01]) (see Table A3 in the supplementary material for full results). When comparing birth cohorts, those in the middle birth cohort perceived somewhat less support than individuals from the youngest birth cohort (Table 2, Fig. 2b).

**Table 2:** Group differences in social support indicators

	Everyday support network			Major support network			Perceived support		
	*IRR*	*95% CI*	*p*	*IRR*	*95% CI*	*p*	*b*	*95% CI*	*p*
Identity (L/G = ref.)									
Bisexual	0.90	[0.75, 1.07]	.220	0.91	[0.75, 1.11]	.342	− 0.19	[− 0.41, 0.02]	.081
Something else	0.82	[0.67, 1.01]	.066	0.88	[0.65, 1.19]	.392	− 0.16	[− 0.39, 0.07]	.175
Changed labels	0.95	[0.76, 1.19]	.679	0.96	[0.78, 1.18]	.706	− 0.02	[− 0.25, 0.21]	.897
Constant	6.40	[5.63, 7.26]		3.48	[3.03, 3.99]		0.08	[− 0.04, 0.21]	
Cohort (Younger = ref.)									
Middle	0.85	[0.72, 1.00]	.055	1.09	[0.85, 1.39]	.505	− 0.27	[− 0.50, − 0.04]	.020
Older	0.98	[0.79, 1.21]	.870	1.15	[0.97, 1.37]	.115	− 0.12	[− 0.29, 0.06]	.194
Constant	6.24	[5.65, 6.90]		3.17	[2.86, 3.52]		0.08	[− 0.03, 0.20]	
Observations (respondents)	1412	(706)		1412	(706)		1412	(706)	

MI data, weighted. Support networks regressed on sexual identity using negative binomial regressions, effects presented as incidence-rate ratios. Perceived support networks regressed on sexual identity using linear regression, results y-standardized. Standard errors clustered by individual

### Aim 2: Effects of Social Support on Well-Being

#### Main Effects of Social Support on Well-Being

A summary of main effects of social support on well-being is provided in Table 3. Only results for overall social support measures are reported, as analyses on the subscales for family and friends showed similar results. In terms of model building, we initially estimated effects of everyday and major social support networks on well-being bi- and tri-variatly, adding perceived social support in a last step. This way, we acknowledged that perceptions of social support could be endogenous to the size of respondents’ social support networks (Berkman et al., 2000; Thoits, 2011). The sizes of respondents’ social support networks were minimally predictive of any of the outcome measures. In the fixed effects models, the largest effect size estimate in expected direction was 0.04 (found for the effect of major social support network size on life satisfaction), indicating that enlarging a social support network by one person was expected to increase a respondents’ well-being by 0.04 SDs at best. Estimates of the effects of support network size were close to zero in random effects analyses too, albeit slightly larger than effects estimates from the fixed effects analysis.

**Table 3:** Associations between social support and well-being

	Fixed effects											
	*b*	*95% CI*	*p*	*b*	*95% CI*	*p*	*b*	*95% CI*	*p*	*b*	*95% CI*	*p*
*K6*												
Everyday support network	0.01	[− .01, 0.03]	.469				0.01	[− .01, 0.03]	.378	0.01	[− .01, .03]	.364
Major support network				− 0.01	[− .03, 0.02]	.707	− 0.01	[− 0.04, 0.02]	.534	− 0.01	[− 0.04, 0.02]	.539
Perceived support										− 0.02	[− 0.11, 0.08]	.753
*Cantril*												
Everyday support network	0.01	[− 0.02, 0.03]	.907				0.00	[− 0.02, 0.03]	.761	0.00	[− 0.02, 0.03]	.792
Major support network				− 0.01	[− 0.04, 0.02]	.549	− 0.01	[− 0.05, 0.02]	.502	− 0.01	[− 0.05, 0.02]	.493
Perceived support										0.02	[− 0.10, 0.13]	.793
*Life satisfaction*												
Everyday support network	0.02	[− 0.01, 0.04]	.598				0.03	[− 0.02, 0.04]	.499	0.01	[− 0.02, 0.03]	.667
Major support network				− 0.02	[− 0.00, 0.08]	.667	− 0.03	[− 0.01, 0.08]	.523	0.04	[− 0.01, 0.08]	.129
Perceived support										0.11	[0.01, 0.21]	.032
Random effects												
*K6*												
Everyday support network	− 0.01	[− 0.03, 0.00]	.067				− 0.01	[− 0.02, 0.01]	.322	− 0.00	[− 0.02, 0.01]	.660
Major support network				− 0.03	[− 0.05, − 0.01]	.010	− 0.02	[− 0.05, − 0.00]	.049	− 0.02	[− 0.04, 0.00]	.092
Perceived support										− 0.10	[− 0.17, − 0.03]	.006
*Cantril*												
Everyday support network	0.02	[0.01, 0.04]	.007				0.02	[0.00, 0.03]	.023	0.01	[− 0.01, 0.03]	.177
Major support network				0.02	[− 0.00, 0.04]	.103	0.01	[− 0.02, 0.03]	.509	0.00	[− 0.02, 0.03]	.779
Perceived support										0.16	[0.08, 0.24]	<.001
*Life satisfaction*												
Everyday support network	0.04	[0.02, 0.05]	< .001				0.03	[0.01, .04]	.006	0.02	[− 0.00, 0.03]	.101
Major support network				0.06	[0.03, 0.09]	< .001	0.04	[0.01, 0.08]	.013	0.03	[− 0.00, 0.07]	.051
Perceived support										0.24	[0.16, 0.32]	<.001
Observations	1412											

MI data, weighted. Results y-standardized

For the effect of perceived social support on well-being outcomes, effect estimates were substantially higher in random effects than in fixed effects models. As the random effects model picks up between-person differences that the fixed effects model filters out, this means that although people with higher levels of perceived social support also reported better well-being, within-person changes in social support over time did not contribute to improved well-being. In the fixed effects analyses, perceived social support had practically no effect on psychological distress (K6) and happiness (Table 3), but had a modest effect on life satisfaction (*b* = − 0.11, 95% CI [0.01, 0.21]).

#### Group Differences in the Effects of Social Support on Well-Being

**Fig. 3:**
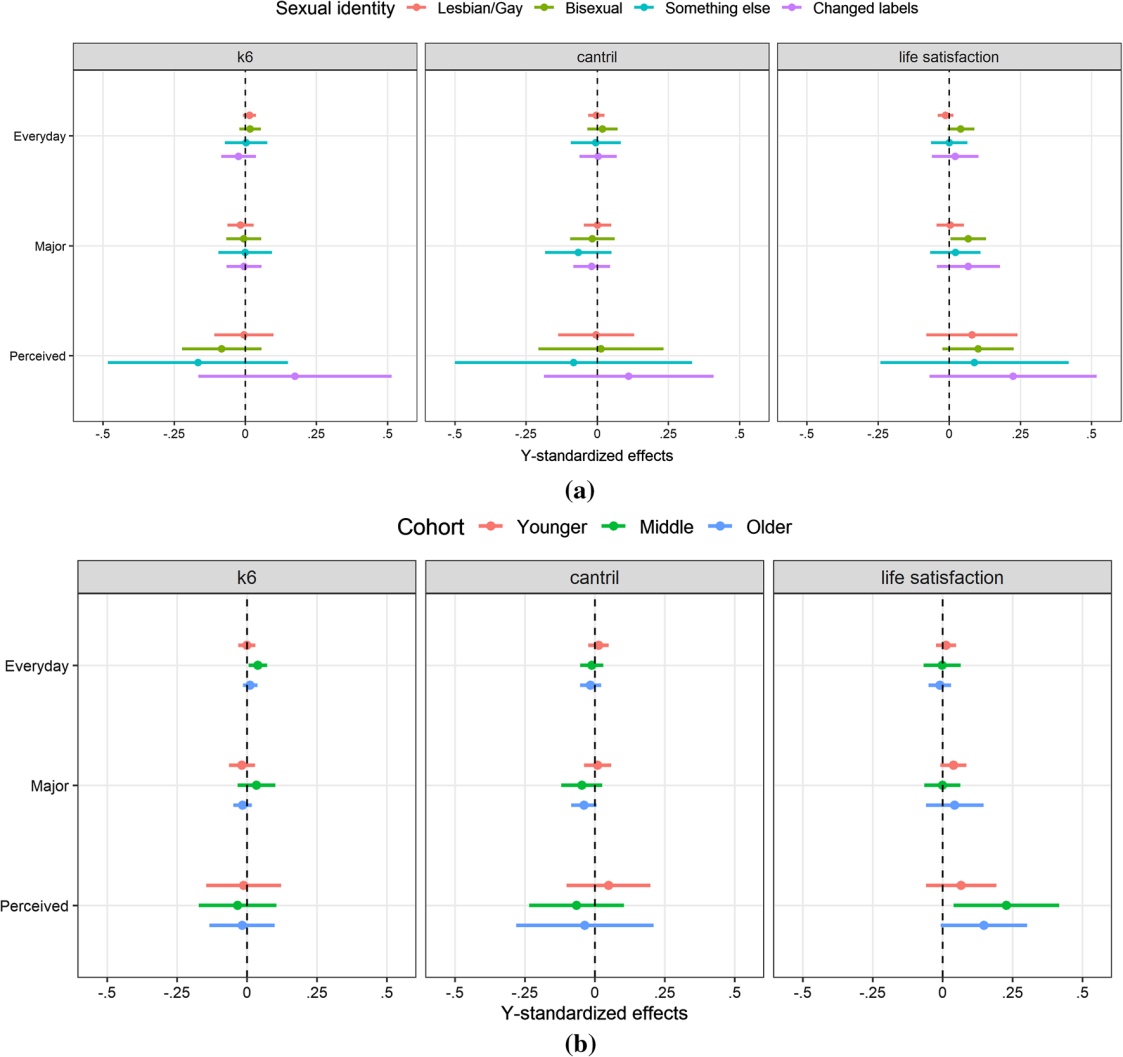
**a** Conditional fixed effects and 95% confidence intervals by sexual orientation of social support on well-being. **b** Conditional fixed effects and 95% confidence intervals by birth cohort of social support on well-being. *Note*: Estimates come from a linear fixed effects regression without covariates. MI data, weighted

Figure 3 provides a summary of effects of social support on well-being by sexual orientation group and birth cohort in a fixed effects regression. Conditional fixed effects were obtained by interacting social support measures with sexual identity. We report effects for overall social support network size and overall perceived social support, as analyses employing family and friends subscales showed similar results. Conditional effects were small across both sexual identity groups and birth cohorts, for all measures of well-being. Furthermore, none of the conditional effects across sexual identity groups or birth cohorts differed significantly from each other. In other words, conditional effects within sexual identity groups and birth cohorts looked very much like main effects.

### Additional Analyses

We ran two sets of additional analyses to corroborate conclusions regarding Aim 2. To account for potential confounding by time-varying variables, we ran both fixed and random effects regressions after including covariates that could worsen people’s access to social support and have detrimental effects on well-being: social and psychological (minority) stressors, whether respondents had health insurance (a proxy for economic status), romantic relationship status, and physical health (details on the exact operationalization of these measures and a summary of the results of models controlling for these time-varying covariates is provided in the Supplementary materials). As controlling for these variables did not lead to substantively different conclusions, we only present effects of models without covariates for parsimony.

Second, to explore whether the relation between social support and well-being was subject to reverse causality, we conducted random-intercept cross-lagged panel models, which simultaneously isolate within-person change from between-person differences and estimate cross-lagged effects (Hamaker et al., 2015). As this method requires three waves of data, this analysis could only be conducted for perceived social support (measured waves 1–3) through extending the analytical sample with wave 1 observations, not for support networks (measured waves 2 and 3 only). In these analyses, we did not detect substantial effects of social support on well-being, nor reverse effects. We also did not detect substantial sexual identity or birth cohort differences in these effects, which were tested using multiple group analysis (Mulder & Hamaker, 2021). All in all, these additional analyses corroborated results from our main analyses.

## Discussion

Using data from a longitudinal USA national probability sample, this article described the levels of social support among three birth cohorts of sexual minority individuals and assessed differences in social support by sexual identity and birth cohort. Furthermore, we tested the association between social support and well-being and examined sexual identity and birth cohort differences in this association.

Regarding the first, descriptive aim of this study, conclusions were twofold. On the one hand, substantial between-group differences were found in the composition of both everyday and major social support networks in terms of family and friendship ties. With regard to sexual identity, bisexual people relied on fewer friends for social support. Furthermore, people from younger birth cohorts relied on more family members than respondents from the oldest birth cohort.

On the other hand, these differences in composition are not reflected in our analyses, and we detected only small group differences in overall sizes of support networks, as well as limited group differences in perceived social support. Regarding sexual identity, these findings are in line with an image of humans as resourceful individuals (Lindenberg, 2001): Non-LG sexual minority individuals seem able to fulfill needs for social support to a similar extent as lesbian and gay individuals, even in light of the obstacles that their marginalized position in both the LGBT community and society at large might pose. Regarding birth cohort, these findings suggest that the increased societal acceptance of sexual diversity has opened opportunities of fulfilling social support needs for both young and old sexual minority individuals, leading to similar levels of social support in both younger and older birth cohorts. In line with this claim, recent research shows that older LGBT adults have social networks of similar size and engage in social contact to a similar extent as heterosexual older adults (Hsieh & Wong, 2020). From a more pessimistic point of view, however, these results could also imply that the promotion and securement of the legal rights of the LGB population has not been accompanied with equally large diminishments in minority stress in the daily lives of sexual minority individuals (Liu & Reczek, 2021), leading to difficulties in accessing sufficient social support for sexual minority individuals from both older and younger birth cohorts.

Our results regarding the effect of social support on well-being are surprising. Although random effects analyses suggested a modest positive effect of perceived social support on well-being, this effect reduced to close to zero in fixed effects analyses. This suggests that the positive association between social support and well-being may be brought about by confounding factors. As revealed by our scan of the literature, the large majority of recent empirical research studying the effect of social support has done so using cross-sectional data. As such, the within-person effect might have been overestimated, in particular if confounders include traits that are difficult to measure (comprehensively). For instance, the seminal work on social support and health by House et al. (1988) highlighted a misanthropic personality as a potential confounder, which represents a hard to measure individual trait that may affect both social support and well-being.

However, as studies using general population samples adopting strict methodological designs (i.e., panel data analysis or (semi-)experimental techniques (e.g., Milner et al., 2016; Wang et al., 2021a, 2021b; West, 2017)) have reported substantial positive effects of social support on well-being, the null findings presented here could suggest that the general measures of social support used in this study are insufficiently tailored to the social support needs of the sexual minority population. That is, their continued marginalized position (Liu & Reczek, 2021; Meyer, 2003) may make sexual minority individuals benefit more from sexuality related support than general social support (Doty et al., 2010), for instance, through alleviating internalized homonegativity and stimulating health sexuality development (Bregman et al., 2013). That said, some recent empirical studies suggest that general social support is actually more important than sexuality related social support in predicting well-being (Sattler et al., 2016; Sheets & Mohr, 2009). Measures of sexuality related social support were not available in the Generations data, leaving an important task for future research to investigate whether sexuality related social support continues to substantially predict well-being among sexual minority individuals when employing rigorous longitudinal designs such as the ones used in this study.

Furthermore, the size of respondents’ social support networks was a poor predictor of well-being and only weakly associated with perceived social support. These findings comprise a specification often the well-known fact that sizes of people’s overall social networks are poor proxies of social support and weak predictors of well-being (Chu et al., 2010; Cohen & Wills, 1985): Even when asking people to select from their social networks those people they can rely on for social support, these support networks relate poorly to perceived social support and well-being. This result has implications for interventions: If one were to stimulate access to social support for sexual minority individuals, it seems more fruitful to improve existing social support relationships than to establish new ones.

### Limitations and Suggestions for Future Research

This study was not without limitations. First, like all studies with a relatively short follow-up period, we cannot clearly distinguish age and cohort effects. The fact, for instance, that members of the youngest birth cohort relied more on family for social support than people in the two older birth cohorts is consistent with a social change cohort explanation: Younger sexual minority people today enjoy better acceptance by family, leading to higher levels of social support from family members than older sexual minority individuals. However, cohort differences in reliance on family for social support might also be a consequence of younger people still being (financially) dependent on parents and perhaps still living with their parents.

Second, that the size of respondents’ social support networks did not predict their well-being could be due to the fact that the introductory text that primed respondents to think of their support ties conflated different types of social support (Berkman et al., 2000; Holt-Lunstad & Uchino, 2015), which may not all be equally important for well-being: The text describing everyday social support networks discussed both instrumental (i.e., help, aid or assistance with tangible needs) and emotional support (i.e., the amount of love, caring, and sympathy available from others), whereas the introductory text on major social support describes both instrumental and appraisal support (i.e., help in decision-making, giving appropriate feedback). As some studies show that emotional and instrumental support may be differentially related to well-being (Fisher et al., 2021), future research should disentangle these different support types when measuring support networks in sexual minority individuals to see if for some types of support, the size of these networks is relevant for well-being.

Third, sample size requirements forced us to combine all respondents not identifying as lesbian, gay or bisexual into one group labeled “something else.” We did not find that this group of respondents differed strongly from LGB respondents in terms of access to social support and the link between social support and well-being. Future research using even larger samples than the one employed here is needed to disentangle whether this means that social support needs and access do not differ systematically between LGB and non-LGB sexual minority individuals, or whether non-LGB sexual minority individuals is a heterogeneous group, whose social support access and needs differs substantially between groups with specific sexual identities.

Relatedly, we exercised caution when operationalizing our sexual identity variable by distinguishing between respondents with stable identity labels and those changing identity labels between waves, labeling the latter group “Changed labels.” However, the individuals placed in this group self-identified themselves using a variety of terms, with only few of them labeling themselves as sexually fluid. Future research is needed to disentangle whether the social support needs of individuals changing identity labels over time and those explicitly defining themselves as sexually fluid differ.

Lastly, the data employed in this study combined two methodological strengths to make a contribution to the literature: longitudinal data and a national probability sample. However, two of our three social support indicators, everyday and major social support network size, were only measured on two occasions. This prevented us from capitalizing on the longitudinal nature of our data in more nuanced ways when testing the effects of these indicators, for instance, to explore whether their link with well-being suffers from reversed causality, was nonlinear in nature, or to test for the presence of lagged effects: Such analyses require (many) more waves of data (Leszczensky & Wolbring, 2022; Singer & Willett, 2003) and a longer observation window. We therefore call upon future research collecting data on support networks and well-being in sexual minority populations to record as many data waves as possible.

### Conclusion

Using national probability data on three birth cohorts of sexual minority individuals from the USA, this study analyzed social support patterns and the effect of social support on well-being, and explored sexual identity and birth cohort differences therein. Despite noticeable differences in the composition of support networks, the sizes of support networks and levels of perceived social support were remarkably similar across sexual identities and birth cohorts. These findings could signal the resourcefulness of individuals, in that non-LG sexual minority individuals fulfill their social support needs similarly as lesbian and gay individuals, despite their marginalized position in both inside and outside the LGBT community. The absence of birth cohort differences suggests that changes in the societal acceptance of sexual diversity have improved access to social support for both younger and older sexual minority individuals.

Furthermore, longitudinal fixed effects analyses showed the effect of support network size and perceived social support on well-being to be close to zero. This could mean that existing cross-sectional research has overestimated the relevance of social support for the well-being of sexual minority individuals, but also that general social support is insufficiently tailored to the support needs of the sexual minority populations. Future research disentangling effects of general and sexuality related social support using rigorous longitudinal designs could elucidate which of these interpretations is most accurate.

## Supplementary Information

Below is the link to the electronic supplementary material.

Supplementary file1 (DOCX 64 kb)

## Data Availability

Data and methodology can be found at: https://www.icpsr.umich.edu/web/DSDR/studies/37166.
