# Supplementary material for: Sexual Identity and Birth Cohort Differences in Social Support and Its Link with Well-Being among Sexual Minority Individuals
Source: Arch Sex Behav. 2022 Aug 18;55(2):769–87. doi: 10.1007/s10508-022-02366-9 (PMC13048958; doi:10.1007/s10508-022-02366-9)
Supplement: Supplementary file 1 — Supplementary file1 (DOCX 64 kb) [file 10508_2022_2366_MOESM1_ESM.docx]

**Online Supplementary**

Table A1. Group differences in family and friendship ties in everyday social support networks

|  | Family ties | | Friendship ties | |
| --- | --- | --- | --- | --- |
|  | IRR | 95% CI | IRR | 95% CI |
| Identity (L/G =ref.) |  |  |  |  |
| *Bisexual* | 1.13 | [0.89,1.42] | 0.78^*^ | [0.64,0.95] |
| *Something else* | 0.81 | [0.54,1.23] | 0.94 | [0.71,1.26] |
| *Changed labels* | 0.79 | [0.58,1.10] | 1.06 | [0.84,1.34] |
| Constant | 1.87 | [1.60,2.18] | 2.83 | [2.48,3.23] |
|  |  |  |  |  |
| Cohort (Younger = ref.) |  |  |  |  |
| *Middle* | 0.75^*^ | [0.59,0.95] | 0.89 | [0.73,1.09] |
| *Older* | 0.78 | [0.59,1.04] | 0.96 | [0.78,1.19] |
| Constant | 2.02 | [1.76,2.31] | 2.75 | [2.45,3.08] |
| Observations | 1412 |  | 1412 |  |

Exponentiated coefficients; 95% confidence intervals in brackets

^*^ *p* < 0.05, ^**^ *p* < 0.01, ^***^ *p* < 0.001

Table A2. Group differences in family and friendship ties in major social support networks

|  | Family ties | | Friendship ties | |
| --- | --- | --- | --- | --- |
|  | IRR | 95% CI | IRR | 95% CI |
| Identity (L/G =ref.) |  |  |  |  |
| *Bisexual* | 1.10 | [0.91,1.33] | 0.76 | [0.54,1.07] |
| *Something else* | 1.13 | [0.82,1.57] | 0.76 | [0.45,1.27] |
| *Changed labels* | 0.93 | [0.74,1.17] | 1.20 | [0.86,1.66] |
| Constant | 1.48 | [1.31,1.68] | 1.17 | [0.97,1.41] |
|  |  |  |  |  |
| Cohort (Younger = ref.) |  |  |  |  |
| *Middle* | 0.76^**^ | [0.62,0.92] | 1.28 | [0.93,1.75] |
| *Older* | 0.68^***^ | [0.56,0.84] | 1.65^***^ | [1.23,2.21] |
| Constant | 1.71 | [1.55,1.89] | 0.94 | [0.76,1.16] |
| Observations | 1412 |  | 1412 |  |

Exponentiated coefficients; 95% confidence intervals in brackets

^*^ *p* < 0.05, ^**^ *p* < 0.01, ^***^ *p* < 0.001

Table A3. Group differences in perceived social support from family and friends

|  | Support from family | | Support from friends | |
| --- | --- | --- | --- | --- |
|  | IRR | 95% CI | IRR | 95% CI |
| Identity (L/G =ref.) |  |  |  |  |
| *Bisexual* | -0.17 | [-0.40,0.05] | -0.30^*^ | [-0.55,-0.06] |
| *Something else* | -0.34^*^ | [-0.68,-0.01] | -0.04 | [-0.31,0.23] |
| *Changed labels* | -0.19 | [-0.49,0.11] | 0.05 | [-0.19,0.29] |
| Constant | 0.12 | [-0.01,0.25] | 0.09 | [-0.04,0.22] |
|  |  |  |  |  |
| Cohort (Younger = ref.) |  |  |  |  |
| *Middle* | -0.27^*^ | [-0.53,-0.00] | -0.26 | [-0.54,0.01] |
| *Older* | -0.19 | [-0.40,0.02] | -0.10 | [-0.27,0.08] |
| Constant | 0.09 | [-0.04,0.21] | 0.07 | [-0.06,0.20] |
| Observations | 1412 |  | 1412 |  |

95% confidence intervals in brackets

^*^ *p* < 0.05, ^**^ *p* < 0.01, ^***^ *p* < 0.001

**Covariates used in additional analyses**

**Minority stressors**

***LGBT neighborhood acceptance.*** Respondents were asked to indicate whether the city or area they lived in was (1) *a good place*, or (0) *not a good place* to live for “(a) Racial and ethnic minorities, (b) Gay, lesbian, or bisexual people, (c) Transgender people, and (d) Immigrants from other countries.” LGBT neighborhood acceptance was defined as the sum of positive responses to b and c.

***Felt stigma*.** Respondents could indicate whether they *strongly disagreed* (1) to *strongly agreed (2)* on the items *“*Most people where I live think less of a person who is LGB”; “Most employers where I live will hire openly LGB people if they are qualified for the job.”; and “Most people where I live would not want someone who is openly LGB to take care of their children”. The scale represents the mean score on those items. Wave 2 *α* (W2 *α*)*_* = .74; Wave 3 *α* (W3 *α*) = .78.

***Stressful life events and perceived stress.*** Operationalized as the total number of times any of 12 stressful life events occurred in the lives of respondents in the past 12 months. Items included “Have you had trouble with your boss or a co-worker?”; “Did you have serious trouble with the police or the law?”; and “Were you unemployed and looking for a job for more than a month?”

***Victimization*** was operationalized as the mean frequency with which respondents indicated to have experienced victimization in the past year. Items included “being hit, beaten, physically attacked, or sexually assaulted.”; and “Someone threatened you with violence”. Response options were (1) *never*, (2) *once*, (3) *twice*, and (4) *three or more times*. W2 *α* = .70; W3 *α* = .71.

***Everyday Discrimination*** (modified from Williams et al., 1997) was operationalized as the mean response on 9 items stating whether respondents between (1) *often* and (4) *never* in their day-to-day life over the past year were, e.g. “threatened or harassed”, “called names or insulted”, or “treated with less respect than other people”. W2 *α* =.91; W3 *α* = .91.

***Internalized homophobia*** (Herek et al., 2009). Respondents could indicate whether they *strongly disagreed* (1) to *strongly agreed (5)* on five items measuring internalized negativity regarding same sex attraction. Items included “I wish I weren’t LGB”, and “I would like to get professional help in order to change my sexual orientation from LGB to straight.”. W2 *α* = .77; W3 *α* = .76.

**Other covariates**

***Not insured.*** Respondents were coded as (1) *Not insured*  versus (0) *Some form of health insurance* when they marked the response option “I currently do not have health insurance” in a question on health insurance and experiences with healthcare.

***Self-rated health.*** This was measured as the sum of large list medical conditions (e.g., asthma, diabetes, Crohn’s disease) that respondents could indicate to suffer from. Somatic complaints (angina, sleep disorder, eating disorder) were excluded from this list to minimize the danger of reversed causality between self-rated health and well-being.

***Relationship status***. Respondents were coded as being in a romantic relationship when they responded affirmatively to the question “Are you currently in a relationship or feel a special commitment to someone?”

**Results additional analyses**

Table A4. Associations between social support and well-being, controlling for time-varying covariates

|  | Fixed effects | | | | | | | | | | | | |
| --- | --- | --- | --- | --- | --- | --- | --- | --- | --- | --- | --- | --- | --- |
| ***K6*** | *b* | *95% CI* | *p* | *b* | *95% CI* | *p* | *b* | *95% CI* | *p* | *b* | *95% CI* | *p* |  |
| Everyday support network | .01 | [-.01, .03] | 0.383 |  |  |  | .01 | [-.01, .03] | 0.267 | .01 | [-.01, .03] | 0.245 |  |
| Major support network |  |  |  | -.01 | [-.03, .02] | 0.594 | -.01 | [-.04, .02] | 0.404 | -.01 | [-.04, .02] | 0.417 |  |
| Perceived support |  |  |  |  |  |  |  |  |  | -.02 | [-.11, .06] | 0.587 |  |
| ***Cantril*** |  |  |  |  |  |  |  |  |  |  |  |  |  |
| Everyday support network | .00 | [-.02, .03] | 0.836 |  |  |  | .01 | [-.02, .03] | 0.666 | .00 | [-.02, .03] | 0.709 |  |
| Major support network |  |  |  | -.01 | [-.04, .02] | 0.516 | -.01 | [-.05, .02] | 0.451 | -.01 | [-.05, .02] | 0.439 |  |
| Perceived support |  |  |  |  |  |  |  |  |  | .02 | [-.08, .13] | 0.675 |  |
| ***Life satisfaction*** |  |  |  |  |  |  |  |  |  |  |  |  |  |
| Everyday support network | .02 | [-.01, .04] | 0.170 |  |  |  | .01 | [-.02, .03] | 0.544 | .00 | [-.02, .03] | 0.731 |  |
| Major support network |  |  |  | .04 | [.00, .09] | 0.039 | .04 | [-.00, .08] | 0.076 | .04 | [-.01, .08] | 0.090 |  |
| Perceived support |  |  |  |  |  |  |  |  |  | .11 | [.02, .19] | 0.018 |  |
|  | Random effects | | | | | | | | | | | | |
| ***K6*** |  |  |  |  |  |  |  |  |  |  |  |  |  |
| Everyday support network | -.00 | [-.01, .01] | 0.600 |  |  |  | .00 | [-.01, .02] | 0.599 | .01 | [-.01, .02] | 0.394 |  |
| Major support network |  |  |  | -.03 | [-.05, -.01] | 0.006 | -.03 | [-.05, -.01] | 0.006 | -.03 | [-.05, -.01] | 0.012 |  |
| Perceived support |  |  |  |  |  |  |  |  |  | -.06 | [-.13, .00] | 0.066 |  |
| ***Cantril*** |  |  |  |  |  |  |  |  |  |  |  |  |  |
| Everyday support network | .01 | [-.00, .03] | 0.111 |  |  |  | .01 | [-.01, .03] | 0.243 | .00 | [-.01, .02] | 0.584 |  |
| Major support network |  |  |  | .02 | [-.01, .04] | 0.151 | .01 | [-.01, .04] | 0.402 | .01 | [-.02, .03] | 0.592 |  |
| Perceived support |  |  |  |  |  |  |  |  |  | .13 | [.04, .20] | 0.003 |  |
| ***Life satisfaction*** |  |  |  |  |  |  |  |  |  |  |  |  |  |
| Everyday support network | .03 | [.01, .04] | 0.000 |  |  |  | .02 | [-.00, .03] | 0.051 | .01 | [-.01, .03] | 0.329 |  |
| Major support network |  |  |  | .06 | [.03, .08] | 0.000 | .05 | [.01, .08] | 0.004 | .04 | [.01, .07] | 0.017 |  |
| Perceived support |  |  |  |  |  |  |  |  |  | .20 | [.13, .27] | 0.000 |  |
|  |  |  |  |  |  |  |  |  |  |  |  |  |  |
| Observations | 1412 | | | | | | | | | | | | |

Notes: MI data, weighted. Results y-standardized. All presented models control for the time-varying covariates listed above

Table A5. Fixed effects regressions of everyday social support network on well-being by sexual identity group, controlling for time-varying covariates

|  | K6 | | Cantril | | Life satisfaction | |
| --- | --- | --- | --- | --- | --- | --- |
|  | *b* | *95% CI* | *b* | *95% CI* | *b* | *95% CI* |
| Lesbian/Gay#everyday support | 0.02 | [-0.00,0.04] | -0.00 | [-0.03,0.02] | -0.01 | [-0.04,0.01] |
| Bisexual#everyday support | 0.02 | [-0.01,0.05] | 0.02 | [-0.03,0.07] | 0.04 | [-0.01,0.08] |
| Something else#everyday support | 0.00 | [-0.07,0.07] | 0.00 | [-0.08,0.09] | -0.00 | [-0.07,0.06] |
| Changed labels#everyday support | -0.03 | [-0.08,0.03] | 0.01 | [-0.05,0.07] | 0.03 | [-0.05,0.10] |
| *N* | 1412 | | | | | |

Notes: MI data, weighted. Results y-standardized. All presented models control for the time-varying covariates listed above, as well as for the main effects of the other two indicators of social support

^*^ *p* < 0.05, ^**^ *p* < 0.01, ^***^ *p* < 0.001

Table A6. Fixed effects regressions of major social support network on well-being by sexual identity group, controlling for time-varying covariates

|  | K6 | | Cantril | | Life satisfaction | |
| --- | --- | --- | --- | --- | --- | --- |
|  | *b* | *95% CI* | *b* | *95% CI* | *b* | *95% CI* |
| Lesbian/Gay#major support | -0.02 | [-0.06,0.03] | -0.00 | [-0.05,0.05] | 0.00 | [-0.04,0.05] |
| Bisexual#everyday support | -0.01 | [-0.06,0.05] | -0.02 | [-0.08,0.04] | 0.07^*^ | [0.01,0.13] |
| Something else# major support | 0.01 | [-0.07,0.10] | -0.08 | [-0.19,0.03] | 0.01 | [-0.07,0.10] |
| Changed labels# major support | -0.02 | [-0.07,0.04] | -0.02 | [-0.09,0.06] | 0.08 | [-0.02,0.19] |
| *N* | 1412 | | | | | |

Notes: MI data, weighted. Results y-standardized. All presented models control for the time-varying covariates listed above, as well as for the main effects of the other two indicators of social support

^*^ *p* < 0.05, ^**^ *p* < 0.01, ^***^ *p* < 0.001

Table A7. Fixed effects regressions of perceived social support on well-being by sexual identity group, controlling for time-varying covariates

|  | K6 | | Cantril | | Life satisfaction | |
| --- | --- | --- | --- | --- | --- | --- |
|  | *b* | *95% CI* | *b* | *95% CI* | *b* | *95% CI* |
| Lesbian/Gay#perceived support | -0.03 | [-0.12,0.07] | 0.01 | [-0.11,0.14] | 0.09 | [-0.04,0.21] |
| Bisexual#perceived support | -0.10 | [-0.22,0.03] | 0.03 | [-0.17,0.22] | 0.10 | [-0.02,0.23] |
| Something else# perceived support | -0.12 | [-0.41,0.17] | -0.10 | [-0.49,0.29] | 0.04 | [-0.29,0.37] |
| Changed labels# perceived support | 0.20 | [-0.10,0.50] | 0.08 | [-0.22,0.38] | 0.20 | [-0.10,0.50] |
| *N* | 1412 | | | | | |

Notes: MI data, weighted. Results y-standardized. All presented models control for the time-varying covariates listed above, as well as for the main effects of the other two indicators of social support

^*^ *p* < 0.05, ^**^ *p* < 0.01, ^***^ *p* < 0.001

Table A8. Fixed effects regressions of perceived social support on well-being by birth cohort, controlling for time-varying covariates

|  | K6 | | Cantril | | Life satisfaction | |
| --- | --- | --- | --- | --- | --- | --- |
|  | *b* | *95% CI* | *b* | *95% CI* | *b* | *95% CI* |
| Younger#everyday support | 0.01 | [-0.02,0.03] | 0.01 | [-0.02,0.05] | 0.01 | [-0.03,0.04] |
| Middle#everyday support | 0.04^*^ | [0.00,0.07] | -0.01 | [-0.04,0.03] | 0.00 | [-0.05,0.05] |
| Older#everyday support | 0.01 | [-0.02,0.04] | -0.01 | [-0.04,0.02] | -0.01 | [-0.05,0.03] |
| *N* | 1412 | | | | | |

Notes: MI data, weighted. Results y-standardized. All presented models control for the time-varying covariates listed above, as well as for the main effects of the other two indicators of social support

^*^ *p* < 0.05, ^**^ *p* < 0.01, ^***^ *p* < 0.001

Table A9. Fixed effects regressions of perceived social support on well-being by birth cohort, controlling for time-varying covariates

|  | K6 | | Cantril | | Life satisfaction | |
| --- | --- | --- | --- | --- | --- | --- |
|  | *b* | *95% CI* | *b* | *95% CI* | *b* | *95% CI* |
| Younger#major support | -0.02 | [-0.06,0.03] | 0.01 | [-0.04,0.05] | 0.04 | [-0.01,0.08] |
| Middle#major support | 0.02 | [-0.05,0.10] | -0.04 | [-0.12,0.05] | 0.02 | [-0.05,0.08] |
| Older#major support | -0.02 | [-0.05,0.01] | -0.04 | [-0.08,0.00] | 0.05 | [-0.05,0.15] |
| *N* | 1412 | | | | | |

Notes: MI data, weighted. Results y-standardized. All presented models control for the time-varying covariates listed above, as well as for the main effects of the other two indicators of social support

^*^ *p* < 0.05, ^**^ *p* < 0.01, ^***^ *p* < 0.001

Table A10. Fixed effects regressions of perceived social support on well-being by birth cohort, controlling for time-varying covariates

|  | K6 | | Cantril | | Life satisfaction | |
| --- | --- | --- | --- | --- | --- | --- |
|  | *b* | *95% CI* | *b* | *95% CI* | *b* | *95% CI* |
| Younger#perceived support | -0.02 | [-0.14,0.10] | 0.06 | [-0.08,0.19] | 0.07 | [-0.04,0.18] |
| Middle#perceived support | -0.02 | [-0.16,0.12] | -0.07 | [-0.24,0.11] | 0.18^*^ | [0.03,0.34] |
| Older#perceived support | -0.05 | [-0.15,0.05] | -0.02 | [-0.21,0.17] | 0.16 | [-0.02,0.35] |
| *N* | 1412 | | | | | |

Notes: MI data, weighted. Results y-standardized. All presented models control for the time-varying covariates listed above, as well as for the main effects of the other two indicators of social support

^*^ *p* < 0.05, ^**^ *p* < 0.01, ^***^ *p* < 0.001

**References**

Herek, G. M., Gillis, J. R., & Cogan, J. C. (2009). Internalized stigma among sexual minority adults: Insights from a social psychological perspective. *Journal of Counseling Psychology*, *56*(1), 32–43. https://doi.org/10.1037/a0014672

Williams, D. R., Yan Yu, Y., Jackson, J. S., & Anderson, N. B. (1997). Racial differences in physical and mental health: Socio-economic status, stress and discrimination. *Journal of Health Psychology*, *2*(3), 335–351. https://doi.org/10.1177/135910539700200305
